# Three-Dimensional Lung Tumor Microenvironment Modulates Therapeutic Compound Responsiveness *In Vitro* – Implication for Drug Development

**DOI:** 10.1371/journal.pone.0092248

**Published:** 2014-03-17

**Authors:** Jason E. Ekert, Kjell Johnson, Brandy Strake, Jose Pardinas, Stephen Jarantow, Robert Perkinson, David C. Colter

**Affiliations:** 1 Biologics Research, Biotechnology Center of Excellence, Janssen R&D, LLC, Pharmaceutical Companies of Johnson & Johnson, Spring House, Pennsylvania, United States of America; 2 Arbor Analytics, LLC, Ann Arbor, Michigan, United States of America; University of Central Florida, United States of America

## Abstract

Three-dimensional (3D) cell culture is gaining acceptance in response to the need for cellular models that better mimic physiologic tissues. Spheroids are one such 3D model where clusters of cells will undergo self-assembly to form viable, 3D tumor-like structures. However, to date little is known about how spheroid biology compares to that of the more traditional and widely utilized 2D monolayer cultures. Therefore, the goal of this study was to characterize the phenotypic and functional differences between lung tumor cells grown as 2D monolayer cultures, versus cells grown as 3D spheroids. Eight lung tumor cell lines, displaying varying levels of epidermal growth factor receptor (EGFR) and cMET protein expression, were used to develop a 3D spheroid cell culture model using low attachment U-bottom plates. The 3D spheroids were compared with cells grown in monolayer for 1) EGFR and cMET receptor expression, as determined by flow cytometry, 2) EGFR and cMET phosphorylation by MSD assay, and 3) cell proliferation in response to epidermal growth factor (EGF) and hepatocyte growth factor (HGF). In addition, drug responsiveness to EGFR and cMET inhibitors (Erlotinib, Crizotinib, Cetuximab [Erbitux] and Onartuzumab [MetMab]) was evaluated by measuring the extent of cell proliferation and migration. Data showed that EGFR and cMET expression is reduced at day four of untreated spheroid culture compared to monolayer. Basal phosphorylation of EGFR and cMET was higher in spheroids compared to monolayer cultures. Spheroids showed reduced EGFR and cMET phosphorylation when stimulated with ligand compared to 2D cultures. Spheroids showed an altered cell proliferation response to HGF, as well as to EGFR and cMET inhibitors, compared to monolayer cultures. Finally, spheroid cultures showed exceptional utility in a cell migration assay. Overall, the 3D spheroid culture changed the cellular response to drugs and growth factors and may more accurately mimic the natural tumor microenvironment.

## Introduction

Over the past decade, the rate of discovery of potential therapeutic anti-cancer compounds has expanded, yet their ultimate introduction into the market remains hampered, with a clinical development success rate of approximately 10% [1], [2]. The two main causes for this high attrition rate are low clinical efficacy and/or intolerable toxicity [3], [4]. Unfortunately, drug failures are often not identified until late in development. Therefore, the earlier identification of ineffective and toxic molecules may serve to improve the overall drug discovery process by reducing costs and increasing pipeline quality. Achieving drug approval is very costly (typically ∼1 billion US dollars) [5], [6]. Consequently, it would be advantageous to eliminate compounds that are possibly ineffective before clinical trials and, preferably, before animal testing has started. Improving *in vitro* cell-based assay methods may allow for a more informed forecast of drug candidate efficacy and safety, and thereby eliminate inadequate functioning compounds, while advancing more promising candidates [5], [7]. In order to reduce drug attrition rates and development expenditures, new and more predictive *in vitro* screening assays must be developed.

To achieve this goal it is essential that more complex cellular models that better mimic physiologic tissues within the context of the tumor microenvironment be developed. This could be achieved through 3D cell culture techniques. Cellular functions and responses that occur in tissues are often lost in conventional two-dimensional (2D) cell cultures, limiting the predictive capability of screening assays. When cells are grown in 2D or 3D, there are numerous biological differences that influence how cells might respond to therapeutic compounds [8]–[11]. Ideally, the characteristics of a successful cellular model in cancer biology, for identifying and eliminating compounds, would include reproducibility, scalability, adaptability, and high throughput formats amenable to automation and drug screening.

There are a number of well-recognized and emerging methods that have been used to mimic solid tumors using 3D culture systems. These include, tissue slices or explants, bioreactors using scaffold/microcarriers or hollow fibers, organotypic cultures (multicellular spheroids and cellular multilayers), gel/matrix based cultures [5], [8], [12], [13] and cell printing [14]. Although these systems have several advantages, their utility in drug-screening applications remains a challenge.

Spheroid formation is one of the best characterized models for 3D cell culture and drug screening due to its simplicity, reproducibility, and similarity to physiological tissues compared to other methods [8]. Spheroids are self-assembled clusters of cell colonies cultured in microenvironments where cell-cell interactions dominate over cell-substrate interactions. Several methods have been developed for generating tumor spheroids including spontaneous aggregation, liquid overlaying, hanging drop, spinner flasks, rotary cell culture systems, poly-2-hydroxyethyl methacrylate, low binding plates, gel/matrix based culture, microencapsulation, polymeric scaffolds and micro patterned plates [5], [13], [15], [16]. Many of these methods have limitations such as, reduced spheroid formation, limited culture duration, and disparities in spheroid size.

Oncology drug development has been slow to incorporate 3D spheroid assays into routine drug development studies. However, a recent paper by Vinci *et al* showed convincing evidence that a 3D spheroid based assay is obtainable for use in drug discovery and target validation [17]. This study examined a variety of tumor types and was developed in a microplate environment that has the appropriate and necessary characteristics for drug development.

Many anti-lung tumor drug discovery initiatives currently focus on molecules that target both the EGFR and cMET signaling pathways. This dual targeting strategy has been selected due to the fact that cross talk exits between EGFR and cMET molecular networks [18]–[21]. Elevated EGFR and cMET expression levels correlate with tumor disease progression and are associated with increased tumor growth, cell migration and invasion [22], [23]. EGFR overexpression has been observed in 40% to 80% of non-small cell lung carcinoma (NSCLC) cases and multiple mutations for the EGFR receptor have been described and linked with malignancy. A number of effective EGFR-specific therapeutic compounds against NSCLC, with EGFR activating mutations (exon 19 deletions and L858R point mutations), have been developed and approved for clinical use. These include both small molecule (i.e. Erlotinib and Gefitinib) and antibody (i.e. Cetuximab and Panitumumab) inhibitors. [24]–[26]

Our goal was to develop a more physiologically relevant, high-throughput 3D lung tumor assay to measure compound effects on cellular proliferation and migration utilizing the EGFR and cMET pathways. The initial aim was to compare the differences between a 2D monolayer and 3D spheroid system in the context of lung tumor biology through EGFR and cMET receptor density, and phosphorylation status. We used eight NSCLC cell lines that consistently formed spheroids, have genetically-distinct subtypes, and have various sensitivities to EGFR treatment. However, rather than using traditional 2D cultures, 3D culture systems were implemented to determine the response to compounds stimulated with HGF and the *in vitro* sensitivity to EGFR and cMET inhibitors. We sought to evaluate the effect of the 3D microenvironment on drug responsiveness in terms of cell proliferation using selected clinically validated EGFR and cMET drugs. In addition, we evaluated the efficacy of this panel of four EGFR/cMET pathway inhibitors to block the migration of four of the eight lung tumor cell lines in tumor spheroids.

## Materials and Methods

### Cell lines and culture conditions

For characterization studies, eight tumor cell lines representative of major NSCLC cancer subtypes, (adenocarcinoma and large-cell lung carcinoma of different EGFR and cMET mutational status) were chosen (Table 1). Additional cell lines were tested but were unable to form suitable spheroids. Tumor cell lines were cultured in tissue culture flasks under normal culture conditions (37°C, 5% CO_2_, 95% humidity). All media and supplementation were as suggested by the supplier of the cells (American Type Culture Collection, Manassas, VA, USA). Media were routinely changed two to three times weekly. When subconfluent, cell monolayers were passaged using Accutase (Sigma-Aldrich, St Louis, USA).

**Table 1:** Characteristics of Lung tumor cell lines compared in 3D and 2D culture systems [47].

Cell type	Origin	EGFR	cMet	KRAS	Sensitivity to Erlotinib
H292	Lung squamous cell carcinoma	WT	WT	WT	Sensitive
H1299	Large cell	WT	WT		Insensitive
A549	Lung adenocarcinoma/ bronchioloalveolar	WT	WT	MT G12S	Intermediate
H1993	Lung adenocarcinoma	WT	WT (AMP)	WT	Intermediate
HCC4006	Lung adenocarcinoma	del (L747, S752)	WT	WT	Sensitive
H1650	Lung Bronchiolo-alveolar	del (E746, A750)	WT	WT	Intermediate
HCC2935	Lung adenocarcinoma	del (E746, A750)	WT	WT	Sensitive
H1975	Lung adenocarcinoma	L858R, T790M	WT	WT	Insensitive

All cell lines were shown to be sterile and mycoplasma free.

### Generation of Spheroids

Spheroids were generated by plating lung tumor cells at 1×10^4^ cells/well into “U” bottom Ultra Low Adherence (ULA) 96-well plates (Corning, Tewksbury, USA) at 200 μl/well. These plates stimulate spontaneous formation of a single spheroid of cells within 24 hours (upon incubation at 37°C, 5%CO_2_). Images were captured by Operetta High Content Imaging System (Perkin Elmer, Waltham, USA) using a 2x objective lens and the size was determined using Harmony software (Perkin Elmer). Cell viability at day one and three using CellTiter-Glo Assay (Promega) was performed according to a modified manufacturer's instructions for tumor spheroids [27].

### EGFR and cMET Receptor Expression

#### A) 3D spheroid dissociation of cells

Spheroids were grown for four days, then pooled from 2 and ½ 96 well plates and collected into a 50 ml Falcon tube. Spheroids were centrifuged at 1400 rpm for 5 minutes and washed 2x times with PBS. 10 mls of Cellstripper (Corning) was added and incubated for 1 hour at 37°C. Spheroids were pipetted 5–7 times to dissociate the spheroids. Cells were washed with PBS and resuspended in BD staining buffer (BD Biosciences, San Jose, USA) for receptor staining.

#### B) 2D monolayer dissociation of cells

The day 4 monolayer cultures were washed 2 times with PBS. 100 μls of Cellstripper (Corning) was added to each well and incubated for 40 minutes at 37°C. Cells in the well were pipetted 3–4 times with a 300 μl pipettor and were pooled from 2 and ½ 96 well plates and put into a 50 ml Falcon tube. The cells were washed once in PBS and resuspended in BD staining buffer (BD Biosciences) for receptor staining.

#### C) Flow cytometry staining

Immediately following dissociation the cells grown in a 2D or 3D format were assessed for viability by exclusion of 0.4% Trypan Blue (Life Technologies, Carlsbad, USA). Cell number and viability were calculated using a C-chip hemocytometer (Incyto, Covington, USA). Cells were resuspended at 1×10^6^ cells/mL in BSA Stain Buffer (BD Biosciences), at which point strict adherence to 4°C was followed to minimize receptor internalization. FcRs were blocked with 5 μl per test of Human TruStain FcX™ (BioLegend, San Diego, USA) for 30 minutes at 4°C. 1×105 cells per well were transferred to 96-well V-bottom microplates (Greiner, Monroe, USA). Cells were incubated with three 1∶2 serially diluted concentrations of anti-human HGF R/cMet-PE (R&D systems, Minneapolis, USA) or anti-human EGFR-PE (BioLegend) at empirically determined saturating concentrations. Isotype matched controls (mouse IgG1 Isotype control-PE, R&D Systems and, mouse IgG2bk isotype control-PE, BioLegend) were included at the highest concentration used for the receptor specific mAbs. The F/P ratio of all PE-mAbs were claimed by the manufacturer to be 1∶1. The cells were incubated with antibody for 1-2 hours on ice protected from light. Cells were washed two times with 150 uL of BSA Stain Buffer and resuspended in 250 uL of BSA Stain Buffer containing 1∶50 diluted DRAQ7 live/dead stain (Cell Signaling Technology, Danvers, USA). Single stain controls for PE and DRAQ7 were included. QuantiBRITE PE Beads (BD Biosciences) were used for receptor density determination. Samples were read on either a BD FACSCalibur (BD Biosciences) or Miltenyi MACSQuant flow cytometer (Miltenyi Biotec, Auburn, USA). The data collection channels for PE/DRAQ7 were: FL2/FL4 (FACSCalibur) and B2/B4 (MACSQuant). 1×104 total events were collected for each sample. FlowJo vX software (FlowJo, Ashland, USA) was used for analysis. Live cells were gated according to DRAQ7 exclusion and the geometric mean of the PE fluorescence for the live population was derived. Receptor density values are reported as antibodies bound per cell (ABC). Geometric means for the four QuantiBRITE bead populations were derived in FlowJo. Linear regression was then used to create a standard curve in GraphPad Prism (GraphPad Software, La Jolla, USA) of the log [#of PE molecules/bead] versus the log[geometric mean]. ABC values for the PE-mAbs were interpolated from the Quantibrite standard curve. ABC values calculated for the isotype controls were subtracted from the ABC values from the corresponding EGFR and cMet mAbs.

### Phosphorylation

Cells were released from flasks using Accutase (Sigma-Aldrich) and resuspended in growth media [RPMI 1640 + GlutaMAX (Life Technologies) with 10% FBS (Life Technologies)]. Tumor cells were plated into ULA plates (Corning) or flat bottom culture plates (BD Biosciences) with 1×10^4^ cells per well plated in 200 μl of growth media (5×10^4^ cells/ml). All cells plated in growth media were between passage 2 and 17 and grown for three days. On the day before stimulation the media was changed on cells to wash spheroids/2D plating at least two times with sera free media. After the final wash, the cells were placed in fresh sera free media and incubated overnight.

Serum-free media was carefully removed from the monolayer and from the spheroid cultures to prevent aspiration of cells. Cells were treated with either 100 ng/mL EGF (R&D Systems) or 100 ng/mL HGF (R&D Systems) for 15 minutes. Cells were lysed with MSD lysis buffer and incubated on ice for 30 minutes. Soluble proteins were collected by spinning at 14000 g for 10 minutes. Equal amounts of protein (5 μg; as determined by Pierce BCA Kit [Thermo Scientific, Rockland, USA]) were added to each well of pEGFR (Y1173)/total EGFR (Meso Scale Discovery, Gaithersburg, USA) and pcMET (Y1349)/total cMET (Meso Scale Discovery) MSD plates and assays were performed according to the manufacturer's instructions.

### Combination dosing of EGF and HGF in cell proliferation assay

H1975 lung tumor spheroids and monolayer cultures were generated as described above. On day 1 a combination of EGF and HGF was added to the plates. The concentrations of EGF (R&D Systems) and HGF (R&D Systems) were 250, 125, 62.5, 31.25, 15.63, 10, 7.81, 3.91, 1, and 0 ng/ml. These concentrations were dosed in a 96 well plate with doses of EGF in the columns and doses of HGF in rows. Three plates of this layout were performed for both the U-Bottom (Corning) and Flat Bottom plates types (BD Bioscience). Spheroids and monolayer cultures were incubated with EGF and/or HGF for approximately 48 hrs at which time CellTiter-Glo Assay (Promega, Madison, USA) was performed according to manufacturer's instructions for monolayer cultures and to a modified manufacturer's instructions for tumor spheroids [27].

### Proliferation/Viability Assay

Lung tumor spheroids and monolayer cultures (H292, A549, H1650 and H1975) were generated as described above. Day 3 tumor spheroids were supplemented with 20 ng/ml of HGF (R&D Systems) and treated with EGFR or cMET inhibitors at 1000 nM for Erlotinib, Cetuximab, Crizotinib and MetMab in culture media and applied to cell spheroids or monolayer cultures. IgG_1_ kappa and DMSO (Sigma-Aldrich) (concentration equal to drug-treated cells) were used as vehicle controls. Spheroids and monolayer cultures were incubated with compound for approximately 72 hrs at which time CellTiter-Glo Assay (Promega) was performed according to manufacturer's instructions for monolayer cultures and a modified manufacturer's instructions for tumor spheroids [27]. Growth inhibition due to treatment effect was assessed by normalizing data by dividing by untreated control to create a percentage growth to control. Therefore, a value less than 1 would be growth inhibitory.

### Migration Assay

Round bottom 96-well plates (BD Bioscience) were coated with 0.1% gelatin (EMD Millipore, Billerica, USA) in sterile water for 1 h at 37°C. For compound evaluation studies, day four 1×10^4^ cell tumor spheroids (H292, A549, H1650 and H1975) were transferred to the coated round bottom plates and treated with Erlotinib, Crizotinib, Cetuximab and MetMab in a dilution series with 20 ng/ml of HGF (R&D systems). Controls were treated with vehicle which was either DMSO (Sigma-Aldrich) (concentration equal to drug-treated cells) for Erlotinib and Crizotinib or IgG_1_ kappa (concentration equal to highest drug-treated cells) for Cetuximab and MetMab. Effects of compounds were analyzed at 48 hrs by measuring the area covered by migrating cells using bright field images in a fully automated Operetta high content imaging system (Perkin Elmer) with a 2x objective. Inhibition of cell migration (total area) due to treatment effect was assessed by normalizing data by dividing by media only control to create a percentage cell migration to control. Therefore, a value less than 1 would be inhibitory to cell migration.

### Data analysis and Statistics

Data was represented as mean ± standard error of the mean (SEM), unless otherwise specified. A *P*-value less than 0.05 was considered to indicate statistical significance.

To determine the relationship between concentrations of EGF, HGF and growth, a response surface model was constructed with linear effects for the log-transformed concentrations of EGF and HGF, quadratic effects of the log-transformed EGF and HGF concentrations, and an interaction between the log-transformed EGF and HGF concentrations. In addition, the model included a term for Plate to account for the shifts in growth due to the plate. A value of 1 was added to each concentration to allow the zero concentration point to be included in the analysis.

For compound effects data was analyzed using GraphPad Prism 5 (GraphPad Software, La Jolla, USA) using a point only XY table/Graph with 2 replicate values in side by side columns. Data was subsequently subjected to a log transform using the function X = log(X). Following transformation, data was analyzed for curve fitting using the xy analysis "nonlinear regression curve fit" for sigmoidal dose response with variable slope. EC_50_ values were calculated with Prism 5.

## Results

### Generation and Growth of Spheroids

**Figure 1:**
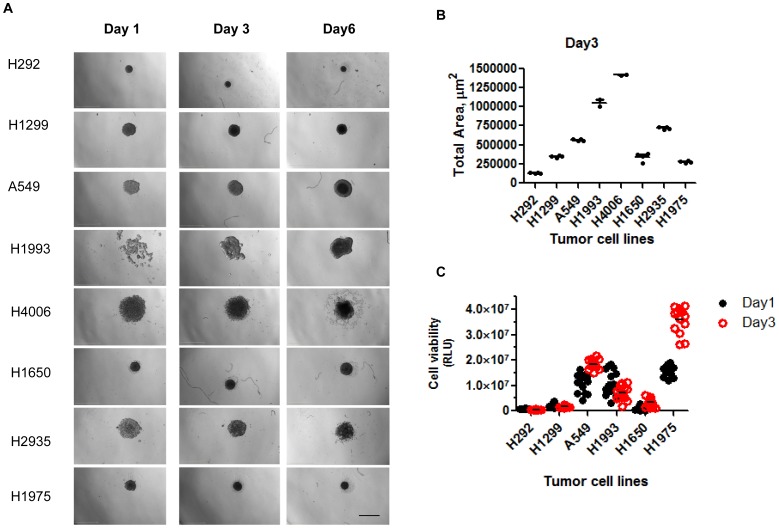
Generation of highly reproducible 3D lung tumor spheroids in culture. A) Eight cell lines were monitored over six days for formation and growth of tumor spheroids. Bright field images were taken daily. Magnification: 2x objective, scan bar: 1mm. B) Total area (μm^2^) of the tumor spheroids at day three were measured using the Operetta imaging system. N is equal to two to five replicates. C) Cell viability was determined at day one and three in six of the eight cell lines by CellTiter-Glo Assay (Promega). N is equal to fourteen replicates.

We evaluated eleven different lung tumor cell lines for their ability to form compact spheroid structures. One EGFR wildtype (H596) and two EGFR mutant (HCC827 and H820) cell lines were unable to form spheroids and were either loose aggregates of cell clusters or irregular, friable aggregates. All of the other eight cell lines were able to form spheroid structures by day 3 in culture (Figure 1A). With the exception of H1993, an EGFR mutant and MET-amplified cell line, all cell lines had formed a spheroid structure by day 1. EGFR wildtype cell line H292 formed the smallest spheroid with a width of approximately 335 μm at day 3 and the largest was EGFR mutant cell line HCC4006 (Figure 1A) with a width of approximately 1135 μm at day 3. HCC4006 and HCC2935 form spheroids, but also had satellite cell structures that were not attached to the spheroid. The lung tumor spheroids generated using the ULA plating method, at 10,000 cells per well, showed strong reproducibility in spheroid size (Figure 1B), with less than or equal to 10% interwell variation in total spheroid area at day 3. 3D spheroids H292, H1299 and H1993 showed no cell proliferation between day 1 and 3 while, A549, H1650 and H1975 had increased cell growth (Figure 1C).

### EGFR and cMET receptor density in lung tumor cell monolayer and 3D spheroid cultures

**Figure 2:**
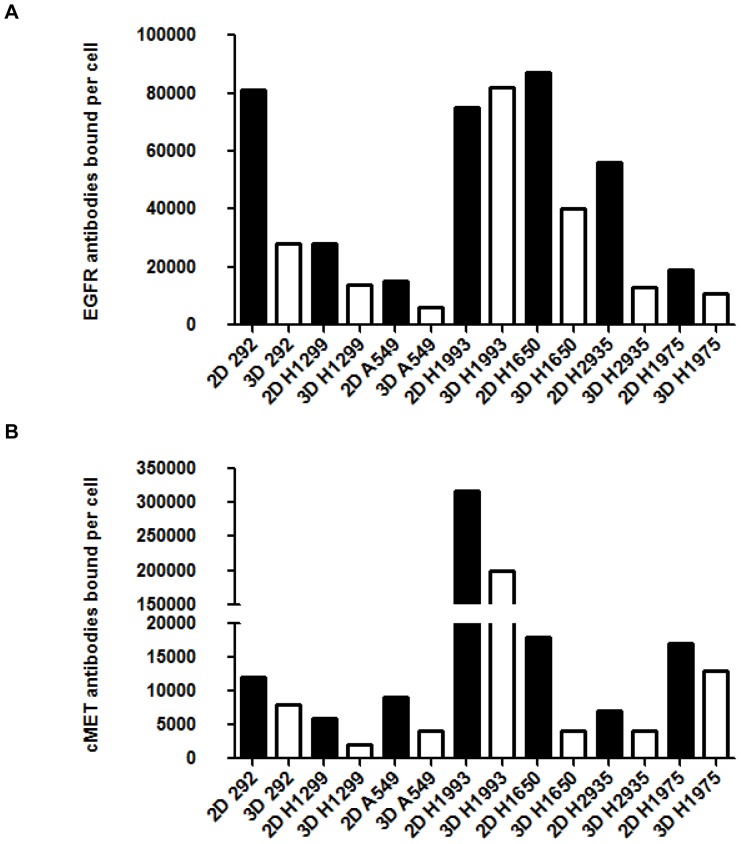
EGFR (A) and cMET (B) receptor density was reduced in 3D spheroid culture compared to 2D monolayer. Day four 2D monolayer cultures and 3D spheroid from seven lung tumor cell lines were measured for EGFR (anti-human EGFR-PE) and cMET (anti-human HGF R-PE) receptor density by flow cytometry. 1×10^4^ total cell events were collected for each sample. Receptor density was determined by using QuantiBRITE PE beads and is representative of two independent experiments.

It has been shown that culture environment can alter receptor expression [10], [28]–[30]. Therefore, the expression of EGFR and cMET on single cells grown either as a monolayer or a tumor spheroid using the ULA plating method was determined by flow cytometry from cells cultured in basal media for four days. A flow cytometry method, using QuantiBRITE beads, was chosen as it can accurately determine the number of EGFR or cMET receptors. [31] Flow cytometry has shown to be highly correlative to other techniques (such as immunofluorescence microscopy and radioligand binding assays) for determining EGFR density [32]. Culturing the cells four days was chosen as all cell lines had formed uniform spheroids and beyond four days cell viability in 3D spheroid cultures decreases. Viability was greater than 50% by Trypan blue (Life Technologies) staining in all cell lines and in both 2D and 3D culture conditions. All lung tumor cells grown either in 2D or 3D expressed EGFR and cMET. Six out of seven cell lines, except for MET-amplified cell line H1993, demonstrated a 1.5 fold or greater decrease in EGFR receptor density in 3D cultures compared to a monolayer determined by the number of EGFR antibodies bound per cell (Figure 2 and S1). EGFR mutant HCC2935 cell line had the greatest difference between 3D and 2D with a 4.3 fold decrease in EGFR receptor density. EGFR wild type and KRAS mutant A549 cells had the lowest EGFR receptor density in both 2D and 3D culture systems while the overall trend in EGFR receptor density between cell lines remained consistent between 2D and 3D.

cMET receptor density was also determined. Six of the seven cell lines had a greater than 1.5 fold decrease in cMET receptor density in the 3D spheroid culture compared to 2D monolayer cultures (Figure 2 and S1). MET-amplified H1993 cell line showed the highest cMET receptor density in both 2D and 3D cultures. The H1299 cells had the lowest cMET receptor density in both 2D and 3D culture.

### Phosphorylation

**Figure 3:**
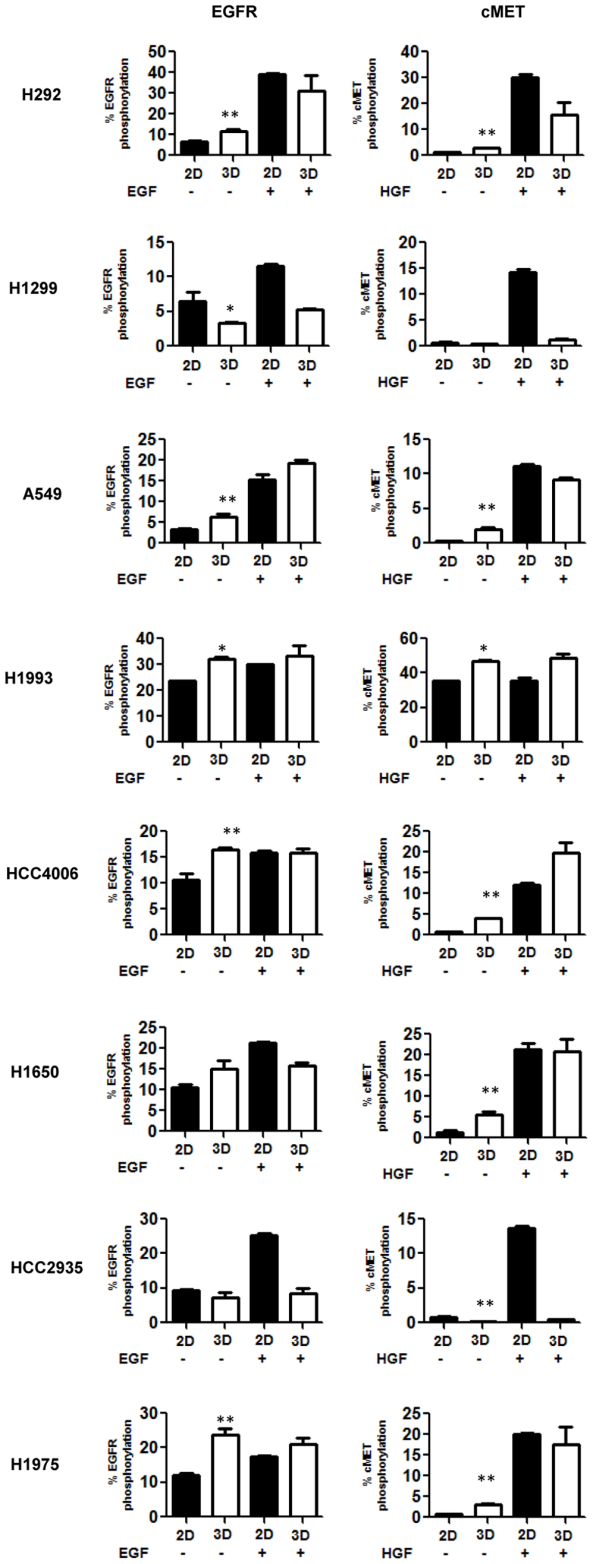
3D tumor spheroid culture alters basal EGFR and cMET phosphorylation and response to ligand stimulation. Day four 2D monolayer cultures and 3D spheroid from eight lung tumor cell lines were stimulated with or without 100/ml of EGF or HGF for 15 minutes. Phosphorylation for EGFR and cMET was determined by MSD assay. N is equal to 4 replicates per condition. T-test comparing basal phosphorylation in 2D verses 3D cultures. (* indicates p<0.05 and ** indicates p<0.005).

Phosphorylation of EGFR and cMET was determined in the monolayer and spheroid cultures by MSD activity in order to determine whether culture microenvironment alters basal levels and intracellular signaling. Total EGFR and cMET, determined by MSD activity, showed the same trends as the receptor density measurements. All eight lung tumor cell lines in 3D spheroids showed reduced receptor expression compared to 2D monolayer cultures (Figure S2). Five of the eight tumor cell lines had significantly (p<0.05) higher basal EGFR phosphorylation levels in the 3D spheroids than the 2D monolayer culture (Figure 3). While H1299 cells had significantly lower levels than 2D monolayers. MET-amplified H1993 cells had the highest basal EGFR phosphorylation levels in both 3D spheroids and 2D monolayer cultures. By contrast the other cell lines in our panel had much lower basal EGFR phosphorylation levels in the 2D monolayer culture.

Six cell lines had significantly (p<0.05) higher basal cMET phosphorylation in the 3D tumor spheroids compared to the monolayer cultures (Figure 3). The HCC2935 cell line which had a low basal EGFR phosphorylation levels had significantly (p<0.005) lower basal cMET levels in the spheroids compared to monolayer cultures. MET-amplified H1993 cells in 2D and 3D cultures had the highest basal cMET phosphorylation (greater than 30%) levels. All of the other cell lines had less than 5% basal cMET phosphorylation in both 2D and 3D cultures.

When monolayer or spheroid cultures were stimulated with EGF or HGF, all of the tumor cell lines, cultured as 3D spheroids, were less responsive to growth factor stimulation (phosphorylation of EGFR and cMET, respectively) than cells grown as 2D monolayers. This was evident when calculating the fold induction over basal values (Figure 3). The average response to EGF across all cell lines in a 2D monolayer was a 2.67 fold increase in EGFR phosphorylation, while there was only a 1.5 fold increase in tumor spheroid cell lines. There was an even greater difference in responsiveness to HGF between 2D and 3D cultures, as the average increase in cMET phosphorylation in all of the cell lines (excluding MET-amplified cell line H1993) was ∼20 fold in 2D monolayer compared to a ∼4 fold increase in 3D spheroid cells. H1993 cells showed no difference in phosphorylation levels after stimulation in both 2D and 3D cells as the cell line is MET-amplified and phosphorylation is HGF-independent.

### Proliferation in response to EGF and HGF stimulation is altered between cells grown as a monolayer or tumor spheroid

**Figure 4:**
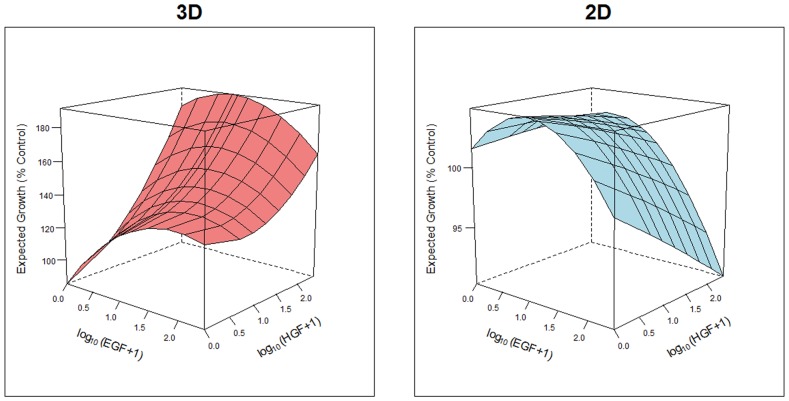
Proliferation response to EGF and HGF is altered in 3D compared to 2D. The plot displays the expected growth surface (i.e. the predicted growth response from the model that was estimated from the data) across varying concentrations of EGF and HGF using the baseline growth estimated for Plate 1 (Figure S3). H1975 cells were plated as a monolayer on a flat bottom plate (2D) or a spheroid in a ULA round bottom plate (3D) and were stimulated with HGF and EGF (250 ng/ml with six serial 1∶2 dilutions) at various combinations at day 1 for 48 hours. These concentrations were dosed in either flat bottom or ULA round bottom plate. Growth (RLU values) was measured in each well by Cell Titer-Glo Assay and was normalized in terms of percent control. The expected growth surface was generated from the linear growth model that included a linear and quadratic terms for EGF and HGF, as well as an interaction term between EGF and HGF.

The proliferation data for this analysis was generated from a combination study using the EGFR mutant H1975 cell line with varying concentrations of EGF and HGF. The concentrations of EGF and HGF were 250, 125, 62.5, 31.25, 15.63, 10, 7.81, 3.91, 1, to 0 ng/ml. The expected growth at the varying concentrations of EGF and HGF was determined (Figure 4). Within 3D spheroid cultures, both EGF and HGF significantly affect changes in growth. The highest concentration of HGF was associated with the highest growth across most concentrations of EGF (Figure 4 and S3). Likewise, the lowest concentration of HGF was generally associated with the lowest growth rate. In addition, as EGF concentration increases in 3D cultures, growth increases, which begins to decrease at higher concentrations. For the 2D monolayer growth data, all EGF terms were significant while no HGF terms (linear or quadratic) were significant. This indicates that EGF significantly affects the growth response in flat bottom plates. Also in the 2D format similar to the 3D, as EGF concentration increases, growth increases, then begins to decrease at higher concentrations (Figure 4 and S3). The most noticeable difference between 2D and 3D was the effect of HGF on cell proliferation, with the highest concentration of HGF in the 2D was associated with the lower growth across most concentrations of EGF. Likewise, the lowest concentration of HGF in 2D was generally associated with the higher growth. This further demonstrates the change in responsiveness of cells when placed into a 3D environment rather than a flat 2D growth surface.

### Effect of EGFR and cMET compounds on cell proliferation in 2D and 3D spheroids

**Figure 5:**
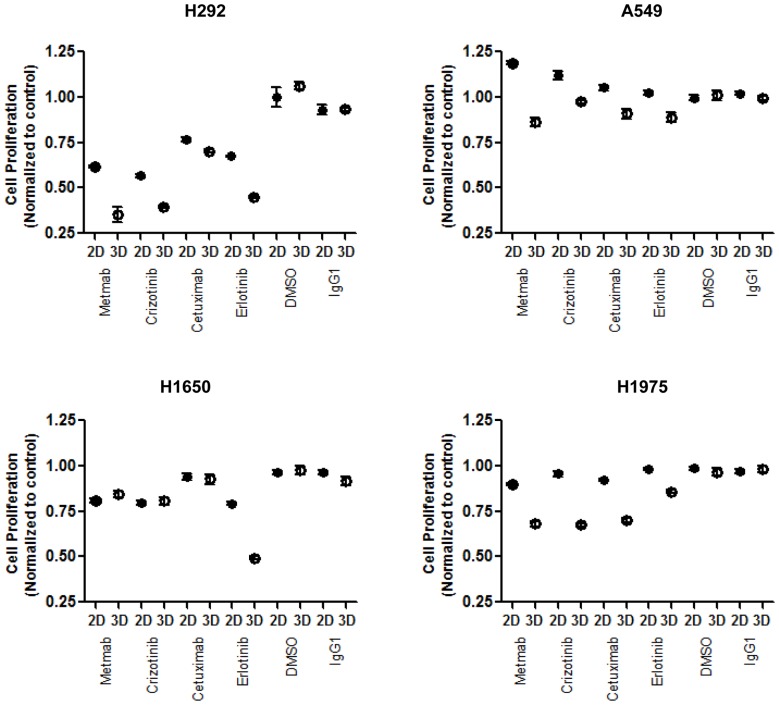
The effects of EGFR and cMET compounds on 2D monolayer and 3D spheroid proliferation. Day 3 lung spheroids or monolayer cultures generated from H292, H1650, H1975 and A549 were treated for 72/ml HGF. IgG_1_ kappa and DMSO were used as vehicle controls. Cell proliferation was determined by CellTiter-Glo Assay (Promega). Cell proliferation (RLU) was normalized to untreated control. Data points represent means + SEM with five replicates and is representative of two independent experiments.

Cell proliferation was evaluated to further characterize the differences in monolayer and spheroid cultures, as well as to assess the impact microenvironment has on cell responsiveness (Figure 5). In H292, H1975 and A549 there was a significant (p<0.05) difference in drug responsiveness for all four compounds in the 3D cultures compared to the monolayer. For H1650, only Erlotinib elicited a significant difference (p<0.05). Data showed that none of the four EGFR or cMET compound inhibitors tested were able to inhibit greater than 50% of cell growth when cells were grown as 2D monolayers. The single arm cMET antibody MetMab had the most significant impact of the four EGFR or cMET compound inhibitors tested, showing a significant (p<0.05) decrease in cell proliferation in H292, H1650 and H1975, cultured as 2D monolayers. However, when cells were grown as 3D spheroids (Figure 5 and S4), MetMab was able to significantly (p<0.05) inhibit growth greater than 25% in H292 and H1975 and Erlotinib inhibited more than 50% in H292 and H1650 cells. EGFR wildtype H292 cells (EGFR wildtype) were the most sensitive to drug treatment (p<0.001) in both 2D and 3D formats. The EGFR mutant cell line H1650 was highly sensitive to MetMab, Crizotinib and Erlotinib (p<0.001), but not Cetuximab in both 2D and 3D formats. When the EGFR mutant cell line H1975 was grown as a monolayer, proliferation was inhibited at greater than 25% for all four drug treatments. However, when the cells were grown as spheroids, they showed elevated sensitivity to Crizotinib, MetMab and Cetuximab (p<0.001). Inhibition of greater than 25% cell growth was not seen in either 2D or 3D formats in EGFR wildtype and KRAS mutant A549 cells. DMSO and IgG isotype controls had no effect on proliferation under both 2D and 3D growth conditions.

### Migration

**Figure 6:**
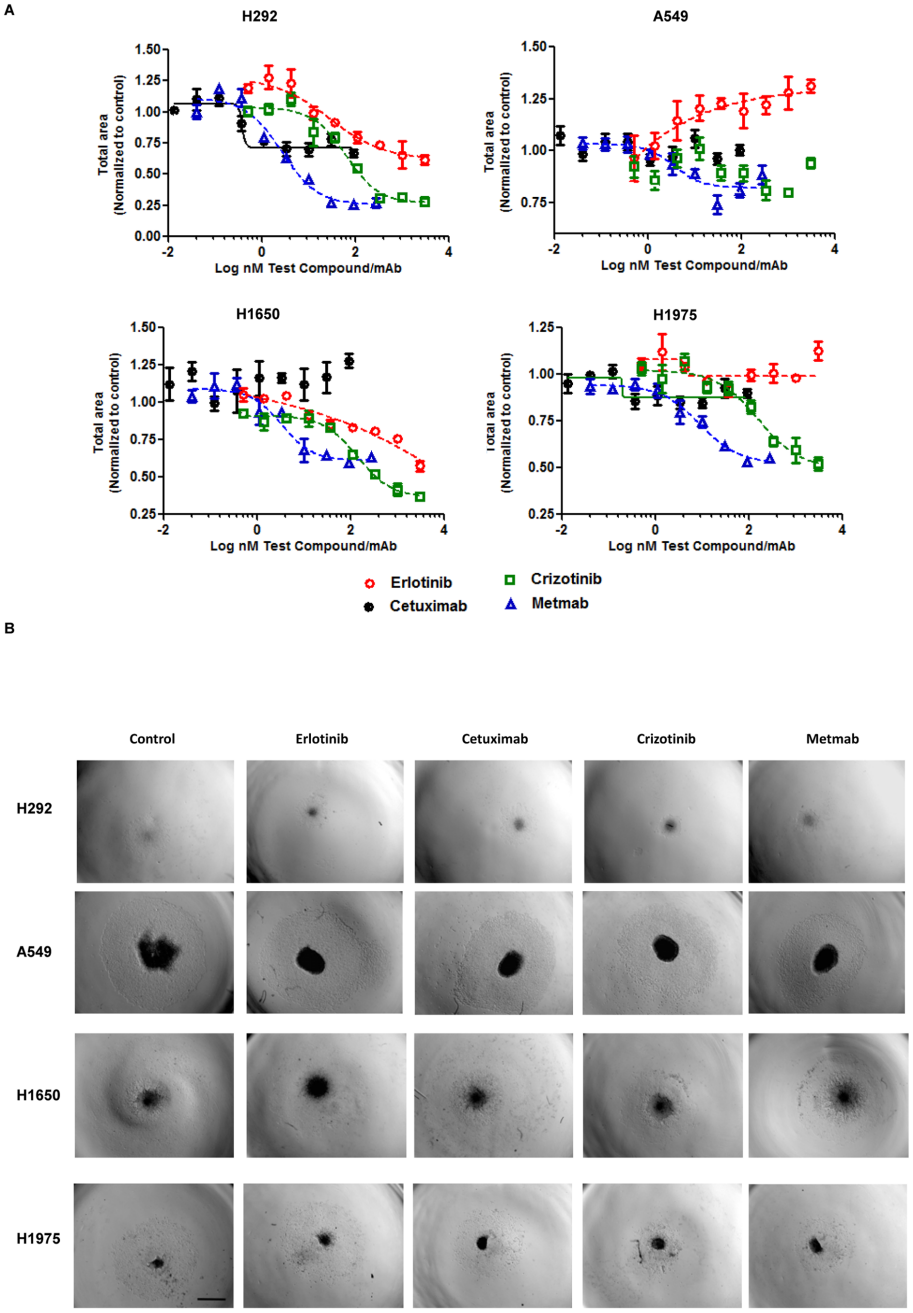
cMET but not EGFR inhibitors reduced cell migration in HGF stimulated lung tumor spheroids. A) Day 4 lung spheroids generated from H292, H1650, H1975 and A549 were treated with Erlotinib, Cetuximab, Crizotinib and MetMab in a dilution series in the presence of 20 ng/ml of HGF for 48 hours to stimulate cell migration. Total area (μm^2^) of migrating and spheroid were determined by using bright field images in a fully automated Operetta high content imaging system (Perkin Elmer). Cell migration (total area) was normalized to media only control to create a percentage cell migration to control. Data are means + SEM with two to five replicates and is representative of two independent experiments. B) Representative bright field images showing drug response after 48 hours in 3D spheroids in cell migration at the highest concentration. Magnification: 2x objective, scan bar 1 mm.

We adapted the tumor spheroid-based migration assay described by Vinci *et al*. [17]. This assay attempts to mimic tumor cell spreading from a solid micro-tumor or micrometastasis. Our adaptations attempt to further improve reproducibility. Tumor spheroids were transferred onto gelatin-coated round bottom 96 well plates (a single spheroid/well) using a multichannel pipette. We elected to use a round bottom plate (as opposed to a flat-bottom plate) as it reduced the incidence of spheroids growing on the edge of the well. Within a few hours, tumor cells could be visualized spreading out from the spheroid and attaching to the gelatin surface. Migration was recorded using the high-throughput Operetta High-Content Analyzer by capturing a z stack and determining the total surface area. Cell migration was stimulated with HGF, as HGF is typically overexpressed in the lung tumor microenvironment [33], [34]. Our data showed minimal cell migration from H292 (wildtype EGFR) spheroids without the addition of HGF (data not shown). In the presence of HGF, EGFR mutant cell line H1650 had the greatest propensity to disseminate from the spheroid followed by A549, H1975 and H292 spheroids. The cell migration pattern in all the tumor spheroids resembled an amoeboid migration pattern in the presence of HGF, while the A549 and H1650 also showed a radial pattern [15] close to the tumor spheroid (Figure 6B). The single arm cMet antibody (MetMab) showed the greatest effect, causing potent dose-dependent inhibition of cell migration in all 4 tumor cell lines (Figure 6A and Table 2). Crizotinib, the small molecule inhibitor for cMet, was also very effective in reducing cell migration in a dose dependent manner in the 4 tumor cell lines (Figure 6A and Table 2). For both Crizotinib and MetMab (cMet inhibitors) a higher EC50 (Table 2) was observed in the mutant EGFR cell lines (H1650 and H1975) compared to the wildtype EGFR cell line H292. Erlotinib (small molecule EGFR inhibitor) caused a modest decrease in H292 and H1650 cell migration but there was no effect in H1975 spheroids. Cetuximab (monoclonal antibody for EGFR) was unable to decrease cell migration and showed no dose dependent decrease in cell migration in all 4 tumor cell lines. All of the EGFR and cMET compounds were unable to inhibit cell migration in the EGFR wildtype and KRAS mutant A549 cells.

**Table 2:** EC50 for EGFR/cMET compounds inhibiting cell migration and cell viability from a 3D lung tumor spheroid.

EC50 nM		Erlotinib	Crizotinib	MetMab	Cetuximab
**H292**	**Total area**	28.4	62.8	2.7	Ambiguous
	**Cell Viability**	163	186	11.33	2.35
**A549**	**Total area**	Ambiguous	Not converged	3.6	Not converged
	**Cell Viability**	202	2909	Ambiguous	Ambiguous
**H1650**	**Total area**	Ambiguous	139	3.4	Not converged
	**Cell Viability**	15.5	174	9.3	3.6
**H1975**	**Total area**	Ambiguous	158.6	8.85	Ambiguous
	**Cell Viability**	Ambiguous	492	80.5	12.3

Total area (μm^2^) of migration pattern and spheroid were determined by using bright field images in a fully automated Operetta high content imaging system (Perkin Elmer). Cell viability (RLU) was determined after cell migration by CellTiter Glo.

**Figure 7:**
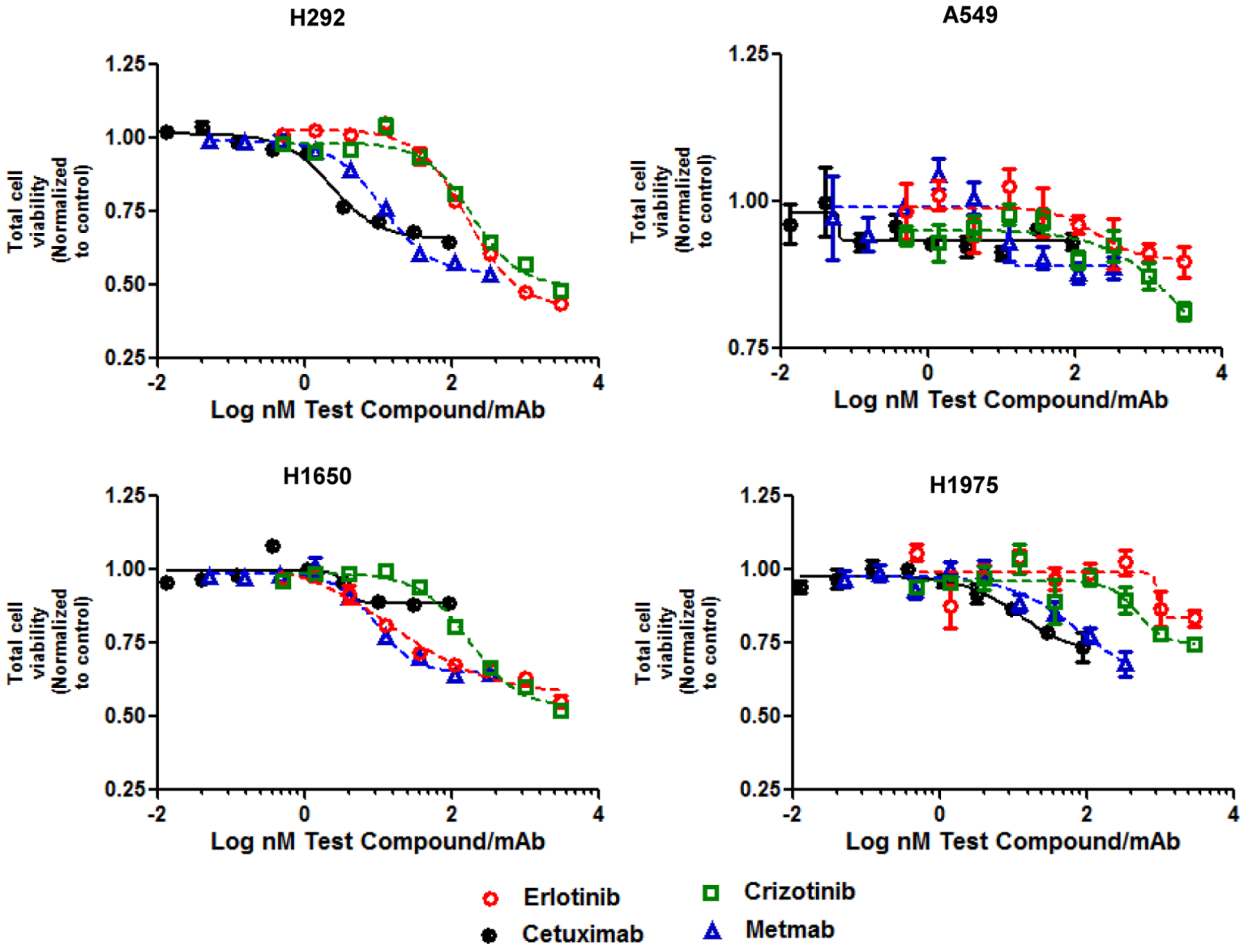
Compounds have an anti-proliferative effect in cell migration assay in HGF stimulated lung tumor spheroids. Day 4 lung spheroids generated from H292, H1650, H1975 and A549 were treated with Erlotinib, Cetuximab, Crizotinib and MetMab in a dilution series in the presence of 20/ml of HGF for 48 hours to stimulate cell migration. Cell proliferation was determined by CellTiter-Glo Assay (Promega). Cell proliferation (RLU) was normalized to untreated control. Data are means + SEM with two to five replicates and is representative of two independent experiments.

After 48 hrs of treatment with EGFR or cMET compounds and measuring cell migration by imaging, cell viability was measured using a modified CellTiter-Glo assay. All of the EGFR and cMET compounds were unable to inhibit cell viability in the EGFR wildtype and KRAS mutant A549 cells (Figure 7). The highest concentration of Cetuximab, Crizotinib and Metmab was required to inhibit mutant EGFR H1975 cells by greater than 25% compared to the untreated control (Figure 7). MetMab and Crizotinib demonstrated an anti-proliferative effect in H292 and H1650 cells (Figure 7). However, the viability EC50 (Table 2) was higher compared to the migration EC50 for each of the cell lines. MetMab and Crizotinib treatment for all cell lines, showed a modest to strong positive relationship between viability and migration (Figure S5). MetMab had a steeper slope (greater anti-proliferative effect) than Crizotinib treated cells for H1975 and H292 cells. Erlotinb showed an anti-proliferative effect in H292 and H1650 (Figure 7) while the viability EC50 was also higher compared to the migration EC50 for each of the cell lines (Table 2). H292 and H1650 cell lines treated with Erlotinib showed a strong positive relationship between viability and migration, but the slope was shallower compared to Metmab and Crizotinib treated cells (Figure S5). Inhibition of cell viability was not affected in H1650 by Cetuximab. H292 cell viability was impacted less by Cetuximab (Figure 7) compared to other compounds but showed a strong positive relationship between viability and migration (Figure S5). Thus, inhibition of cell migration by the EGFR and cMET inhibitors is partially due to their anti-proliferative effects on tumor cells.

## Discussion

To date, a 3D lung tumor model, incorporating multiple lung tumor cell types and specifically targets the EGFR-cMET molecular networks, has not been developed. Additionally, no such model system has ever been validated by comparison to more traditional 2D monolayer culture systems. This study examined whether a 3D lung tumor spheroid culture system could be developed by adapting the Vinci *et al*. spheroid system [17].

The rationale for such a system is based on the fact that some cancer cell lines, in particular lung tumor cell lines, can be grown as small multi-cellular spheroids and other 3D structures. These spheroids may be more representative of solid tumors *in vivo* as they have many features that are similar with cancer such as, similar gradients for oxygen/hypoxia, metabolic requirements, lactate accumulation, and degree of proliferation [17], [35]. Therefore, these spheroid structures may serve as the building blocks for more physiological, *in vitro* tumor model systems - providing more reliable therapeutic readouts compared to 2D assays. We were successful in generating a reliable and reproducible 3D lung spheroid model that rapidly formed spheroids in multiple and diverse lung tumor cell lines. A number of spheroids (A549, H1650 and H9175) demonstrated the ability to proliferate in a 3D spheroid environment which was observed with larger spheroids having increased cell viability. Other cell lines (H292, H1299 and H1993) did not proliferate (no increase or decreased cell viability) with smaller spheroids at day 3 compared to day 1. One of the key goals of this study was to demonstrate the inherent differences between 2D and 3D (Table 3) culture formats.

**Table 3:** Summary of differences between lung tumor cells grown in a 2D monolayer or 3D spheroid culture.

	EGFR and cMET receptor density	Basal Phosphorylation	EGFR and cMET Phosphorylation response to ligands	Cell proliferation in response to EGF and HGF	Inhibition of Proliferation by EGFR and cMET inhibitors	Inhibition to Cell Migration by EGFR and cMET inhibitors
**3D single spheroid compared to 2D monolayer**	Lower	Higher	Lower	High HGF stimulated growth	Increased response	More potent inhibition for compounds in migration assay than in 2D and 3D proliferation assay

We demonstrated that lung tumor cell lines, growing in 3D had lower receptor expression levels for both EGFR and cMET, but higher basal receptor phosphorylation activity compared to 2D monolayer cultures. These data confirm previous studies that have shown altered receptor expression levels in a 3D microenvironment. For example, a recent paper examining colorectal cancer cells grown in 3D, observed a similar phenomenon, where all seven cell lines studies had lower EGFR protein expression compared to 2D monolayer cells [10]. This study also observed elevated phospoMAPK protein levels in the 3D cultured cells, which is downstream of pEGFR. In our study, we observed increased basal phosphorylation of EGFR and cMET in 3D spheroid cultures compared to 2D monolayers. This suggests that phosphorylation of EGFR may be occurring independent of EGF ligand stimulation within 3D spheroids. We only observed MET-amplified H1993 cells to have high basal EGFR and cMET phosphorylation levels in the 2D monolayer system, as these cells are well recognized to be auto-phosphorylated for EGFR and cMET independent of EGF stimulation [36]. This suggests that lung cells, cultured in a 3D environment, may lead to elevated basal phosphorylation, independent of ligand for EGFR and cMET. As a result, this may lead to a decrease in responsiveness to ligand, suggesting a mechanism by which lung tumor cells adapt to their environment, acquiring therapy resistance to EGFR and cMET. This elevated basal phosphorylation of EGFR and cMET could occur through a number of mechanisms. One possibility is through cellular stress which has been shown to occur in 3D spheroid models where lactate and mild hypoxia could be indicative of cellular stress [37]. We observed in a PCR array analysis up-regulation of cellular stress/oxidative stress genes in the 3D lung spheroid cultures compared to the monolayers (data not shown). Cellular stresses, such as TNF-α have been reported to induce the phosphorylation EGFR-Ser1046 via p38 MAPK. This, in turn, may cause EGFR desensitization upon EGF stimulation and endocytosis [38]. Increased local concentrations of HGF in the spheroid may also be leading to autocrine/paracrine effects, as it has been shown that HGF is secreted by tumor cells and could lead to phosphorylation of cMET [39]–[42]. In addition, increased ligand independent cMET phosphorylation could be occurring through alternative mechanisms as well. These possible mechanisms include: constitutive dimerization in the absence of ligand associated with overexpression, pathway activation under hypoxic conditions, and transactivation by other receptors, including EGFR [42].

The change in basal phosphorylation status and reduced responsiveness in the 3D cultures may be linked to the change in cell proliferative response to EGF and HGF in the tumor spheroids. Our data showed that EGF stimulated proliferation within 3D spheroid cultures was reduced compared to that of 2D monolayer cultures. This may be related to the altered phosphorylation status in the 3D cultures. However, we also observed that higher EGF concentrations inhibited growth in both 2D and 3D microenvironments. This is consistent with previous findings [43]. Maximal HGF-induced cell proliferation was also altered in both 2D and 3D, as maximal cell proliferation was induced at low HGF concentrations in 2D, while in 3D cultures, maximal proliferation was observed at high HGF concentrations. Therefore, the observed reduction in cMET phosphorylation in the 3D spheroids could be mechanistically related to this altered proliferation response.

To validate the effectiveness of the 3D spheroid as a lung tumor model for screening EGFR-cMET inhibitors, assays were performed for blocking cell proliferation and migration, in the presence of HGF, with clinically approved EGFR and cMET inhibitors. HGF has been shown to induce EGFR resistance in both wildtype and mutant EGFR lung tumor cells [44]–[46].

To evaluate drug responsiveness of EGFR/cMET inhibitors we employed proliferation and migration assays using four cell lines (H292, A549, H1650 and H1975). Two EGFR wildtype cells lines were chosen (A549 and H292) but A549 is also known to have a KRAS mutation and two EGFR mutant cells were selected (H1650 and H1975). H1975 also carries the extra mutation T790M for EGFR resistance. Each group had cell lines that were more sensitive to EGFR which included H292 and H1650. We chose four different compounds that were EGFR or cMET inhibitors and a small or large molecule and this allowed us to predict the responses. Past reports suggest Erlotinib and Cetuximab should not inhibit proliferation and migration in the A549 and H1975 but H292 and H1650 should be responsive to both EGFR inhibitors [44], [47]–[49]. All cell lines should be responsive to the cMET inhibitors (Crizotinib and MetMab).

Acquired resistance to EGFR-tyrosine kinase inhibitors TKIs is observed in lung cancer with cMET amplification and is considered the main escape route for EGFR-targeted therapies. Tumors with the gatekeeper T790M mutation are frequently found to have overexpression of HGF [33], [34]. A gatekeeper mutation (i.e. T790M second mutation) is the molecular mechanism for acquired resistance. cMET amplification causes upregulation of various tumor cell functions including cell proliferation, survival, cell scattering and motility, epithelial-mesenchymal transition, angiogenesis, invasion and metastasis [50]–[52]. cMET inhibitors have been developed for therapeutic downregulation, which include small molecule (Crizotinib, and SU11274) and antibody (Onartuzumab)[51] therapies. Future therapies are being evaluated that combine EGFR and cMET inhibitors such as combined therapy (i.e. Erlotinib and MetMab) or novel bispecific EGFR/cMET antibody inhibitors [51], [53], [54].

Our data showed that in general, drug responsiveness in the cell proliferation assay was altered, depending on the culture microenvironment. For example, EGFR and cMET inhibitors had greater potency in 3D spheroid cultures compared to 2D monolayer cultures. However, MetMab blocked cell proliferation in both 2D and 3D. This confirms previous findings that showed MetMab inhibition of cell proliferation in BxPC-3 cells [55]. Cetuximab, on the other hand, did not alter cell proliferation in either cell culture microenvironment. This confirms previous reports that many different lung tumor cell lines are resistant to Cetuximab, showing no inhibition in cell proliferation [56]. Another study demonstrated that monolayer proliferation of H292 (wildtype EGFR) was not inhibited by Cetuximab when HGF was added [44].

It has generally been the case that chemotherapeutic agents show increased potency in 2D cell proliferation assays compared to that of 3D spheroid growth assays [57], [58]. This phenomenon of 3D cultured cancer cells, being less responsive or having an altered response to chemotherapeutic drugs, has been demonstrated in multiple studies [59]–[61]. For example Vinci *et al*. [17] showed that tumor cells were less responsive to compounds (17-AAG, PI-103 and CCT130234) in 3D than 2D cultures. However, it was also shown that two cancer cell lines, U-87 and KNS42, were more sensitive to PI-103 in 3D [17], which has been demonstrated by Howes *et al*. as well [62]. Another study showed that there was a low correlation between 2D monolayer verses 3D spheroids in H292 cells based on EC50 for tubulin inhibitors (41 anti-cancer drugs) to inhibit cell proliferation. Most of the anti-cancer agents tested did not show improved potency in the 2D cell proliferation assay compared to 3D spheroid proliferation [35]. Another 3D culture system demonstrated, in studying colorectal cancer EGFR signaling biology, that a significant difference occurred in cell viability, proliferation and gene expression (225 genes altered) between cells grown on 2D tissue culture plates compared to cells grown under 3D laminin- rich-extracellular matrix (lrECM) [10]. KRAS mutant colorectal cells were shown to be unresponsive to EGFR inhibition in 3D compared to 2D [10], and KRAS mutant lung tumor cells have been shown to be resistant to EGFR inhibitors [63], [64]. This was confirmed in our studies with A549 cells being resistant to Erlotinib and Cetuximab in both the proliferation and migration assays. This reveals the importance of evaluating drug responses under suitable cell culture conditions in order to reduce miscalculating the effect of compounds *in vivo*. It also suggests that 3D approaches may better recognize the critical oncogenic pathways and potential targeted inhibitors that may have therapeutic value.

The cell migration assay was a further validation and extension of the 3D spheroid model, as it was effective in discriminating between molecules that inhibited cell migration in different tumor cell lines. HGF has been shown to promote cell migration and invasion [42], [65], [66] and is required in cell migration assays. The EGFR inhibitors were ineffective in blocking cell migration in all of the cell lines (A549, H1975 and H1650) except for EGFR wildtype cell line H292 which was sensitive to EGFR inhibitors. These cell lines are known to be insensitive to EGFR inhibitors [47]. In addition, Erlotinib, but not Cetuximb has been shown to inhibit cell migration [67], which we observed in H292 cells in the spheroid migration assay. MetMab was the most effective inhibitor of cell migration in all cell lines tested. MetMab has been shown (10 μg/ml (100 nM) or higher) to inhibit cell migration in U87 glioblastoma, in a modified Boyden chamber assay, in the presence of 20 ng/mL of HGF [66]. We observed that the EC50 for MetMab for cell migration inhibition in all 4 cell lines was 8 nM or less and maybe a more predictive measure of collective cell migration than isolated single cell migration using the Boyden chamber assay [17], [68], [69]. The EGFR mutant cell lines H1650 and H1975 were less sensitive to the Crizotinib (cMET inhibitor) than the wild type EGFR cell lines H292 and A549. Interestingly, the cMET inhibitors were more potent in the 3D cell migration assay than 2D or 3D cell proliferation assays. We also observed that the inhibition of cell migration was partially due to anti-proliferative effects as we observed reduced cell viability with the EGFR and cMET compounds. There was a modest to strong positive correlation observed between viability and cell migration in the different cell lines with varying compound concentrations. However, the EC50 values for the compounds were higher in the viability assay compared to the migration assay which suggests that the reduced cell migration isn't completely due to the anti-proliferative effects of the compounds. A recent study demonstrated that in lung cancer cells a significant positive correlation between mean proliferation and migration can be observed, and that non-dividing lung cells display slower migration [70]. 3D assay system may therefore have value in helping to elucidate mechanisms and new molecules that could block both proliferation and migration of tumor cells.

Collectively, this is the first detailed analysis of the EGFR-cMET pathways in lung tumor spheroid cultures compared to monolayer cultures. Data showed alterations in protein expression, phosphorylation pattern and responsiveness to EGF and HGF ligand and EGFR and cMET inhibitor compounds (Table 3). These microenvironment induced changes in lung tumor cell physiology add a level of complexity to cell-based assays and may be more representative of the *in vivo* tumor microenvironment. These data demonstrate clear functional differences in tumor cell lines cultured in either 2D or 3D microenvironments. These observations suggest that it may be reasonable to utilize both culture methodologies in drug screening paradigms. However, it is still unclear how close the 3D spheroid microenvironment compares to the actual *in vivo* lung cancer microenvironment. A possible future direction would be to compare these findings to that of primary tumor cell lines, isolated directly from patient lung tumors. This would represent one step closer to physiologically replicating the actual lung tumor microenvironment *in vitro*.

This study shows that 3D spheroid microenvironment alters the cellular response to drugs and growth factors. Based on these findings, it is important that future drug discovery campaigns consider 3D spheroid-based assays as an additional screening tool. These assays may ultimately improve the quality and efficiency of EGFR/cMET targeted drug discovery efforts.

## Supporting Information

Figure S1 **EGFR and cMET receptor expression in lung tumor cell lines grown as a 3D spheroid or monolayer culture.** Cells were grown for four days as either a monolayer culture or spheroid culture (ULA round bottom plates). The cells were removed from the wells and analyzed by flow cytometry for EGFR or cMET expression using PE conjugated monoclonal antibodies. Red depicts 2D monolayer cells and blue is the 3D spheroid cultured cells.
(TIF)

Figure S2 **3D tumor spheroid culture alters total EGFR and cMET.** Total EGFR and cMET was determined by MSD assay in day four 2D monolayer cultures and 3D spheroids from eight lung tumor cell lines. N is equal to 4 replicates per condition.
(TIF)

Figure S3 **Proliferation response to EGF and HGF is altered in 3D compared to 2D.** The plots for 2D (A) and 3D (B) are the growth measurements for the varying concentrations of EGF and HGF by each plate (panels). For this figure, a value of 1 is added to each original concentration value and the augmented concentration value is then transformed to the log10 scale. The y axis is growth which is a RLU value determined by CellTiter Glo after EGF and HGF for two days.
(TIF)

Figure S4 **The effects of EGFR and cMET compounds in 3D spheroid proliferation.** Representative bright field images showing drug response after 72 hours in 3D spheroids in cell proliferation assay. Magnification: 2x objective, scan bar 1mm.
(TIF)

Figure S5 **Positive correlation between cell migration and cell viability in cell migration assay.** The scatterplots by cell type and compound are for log-transformed migration (total area) verses cell viability (RLU value). Total area (μm^2^) of migration pattern and spheroid were determined by using bright field images in a fully automated Operetta high content imaging system (Perkin Elmer). Cell viability (RLU) was determined after cell migration by CellTiter Glo. The r-squared value along with the intercept (a) and slope (b) are shown in the diagrams.
(TIF)
