# Supplementary figures and images for: Three-Dimensional Lung Tumor Microenvironment Modulates Therapeutic Compound Responsiveness In Vitro – Implication for Drug Development
Source: PLoS One. 2014 Mar 17;9(3):e92248. doi: 10.1371/journal.pone.0092248 (PMC3956916; doi:10.1371/journal.pone.0092248)

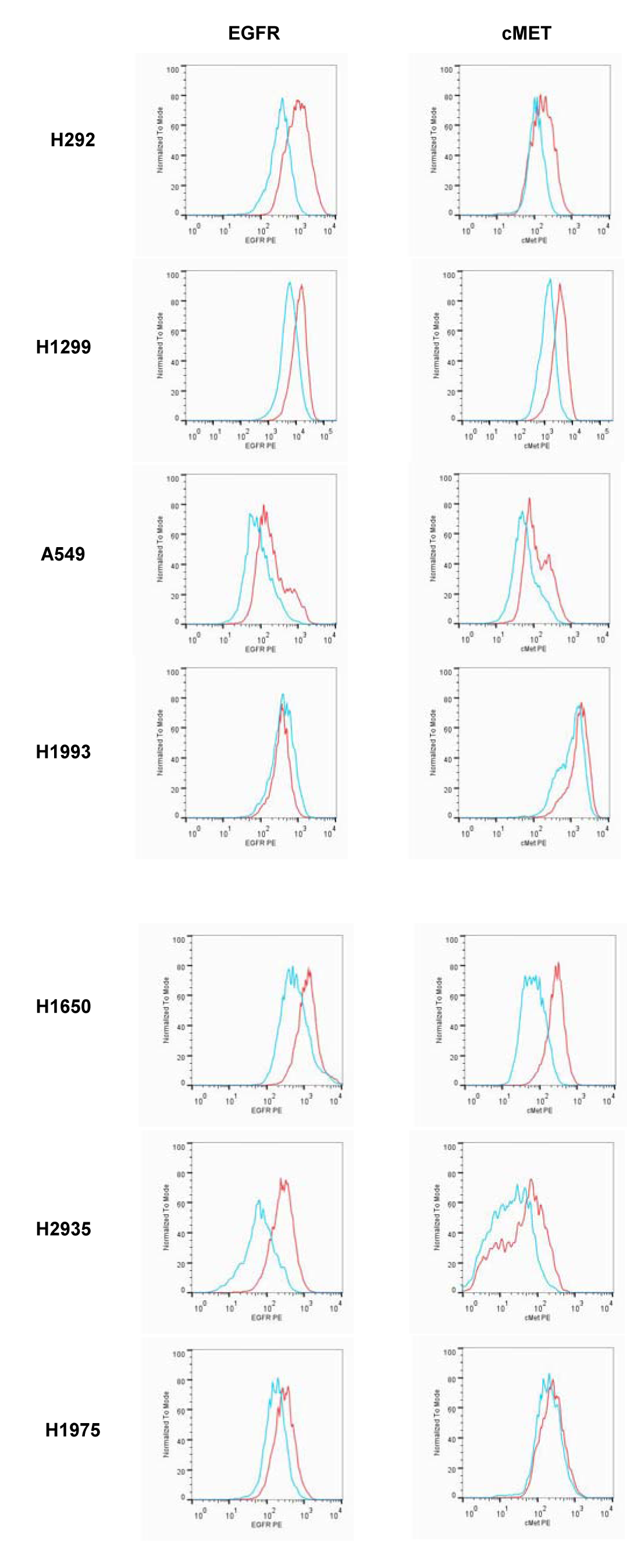

Supplement: Figure S1 — EGFR and cMET receptor expression in lung tumor cell lines grown as a 3D spheroid or monolayer culture. Cells were grown for four days as either a monolayer culture or spheroid culture (ULA round bottom plates). The cells were removed from the wells and analyzed by flow cytometry for EGFR or cMET expression using PE conjugated monoclonal antibodies. Red depicts 2D monolayer cells and blue is the 3D spheroid cultured cells. (TIF) [file pone.0092248.s001.tif]

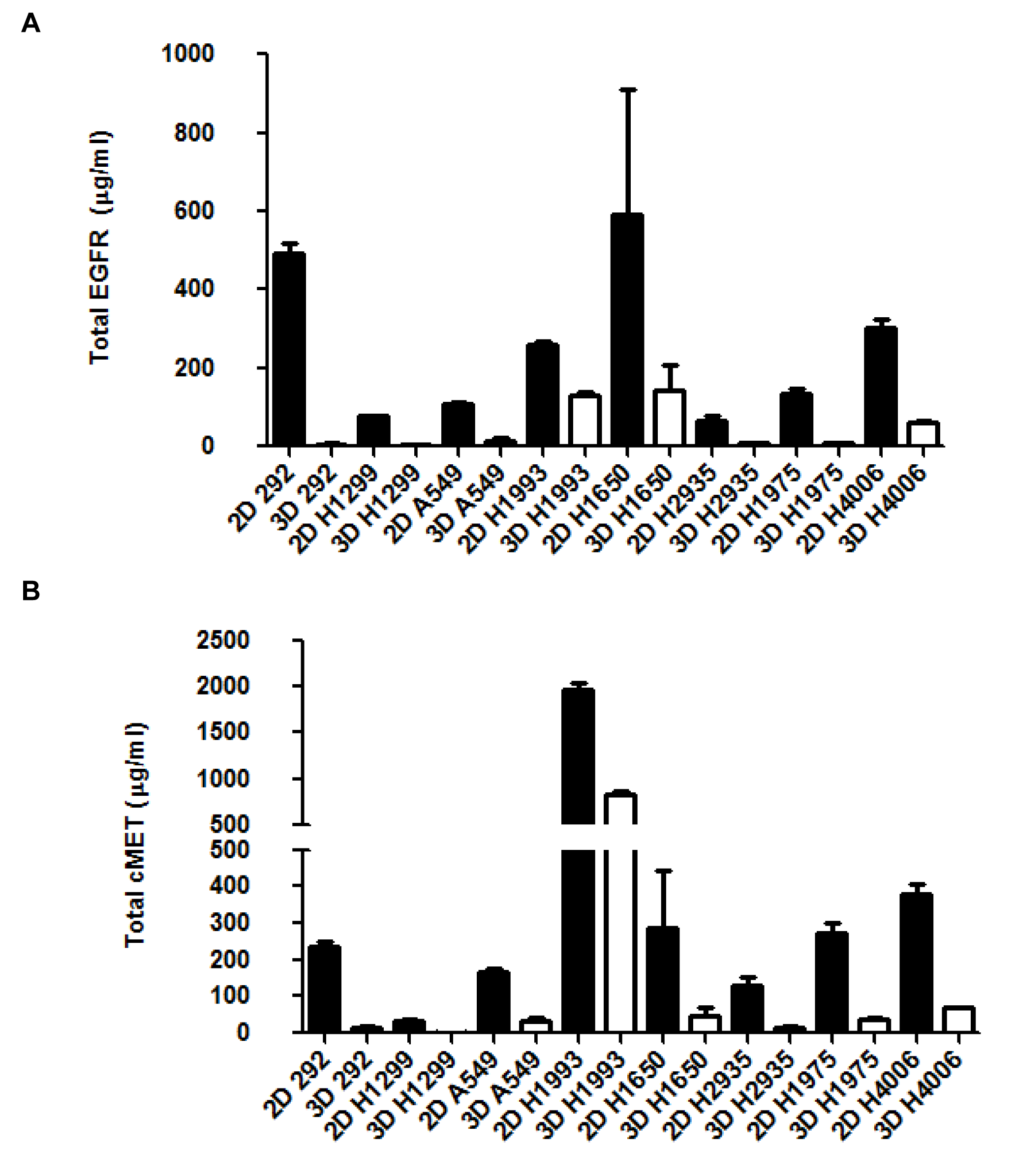

Supplement: Figure S2 — 3D tumor spheroid culture alters total EGFR and cMET. Total EGFR and cMET was determined by MSD assay in day four 2D monolayer cultures and 3D spheroids from eight lung tumor cell lines. N is equal to 4 replicates per condition. (TIF) [file pone.0092248.s002.tif]

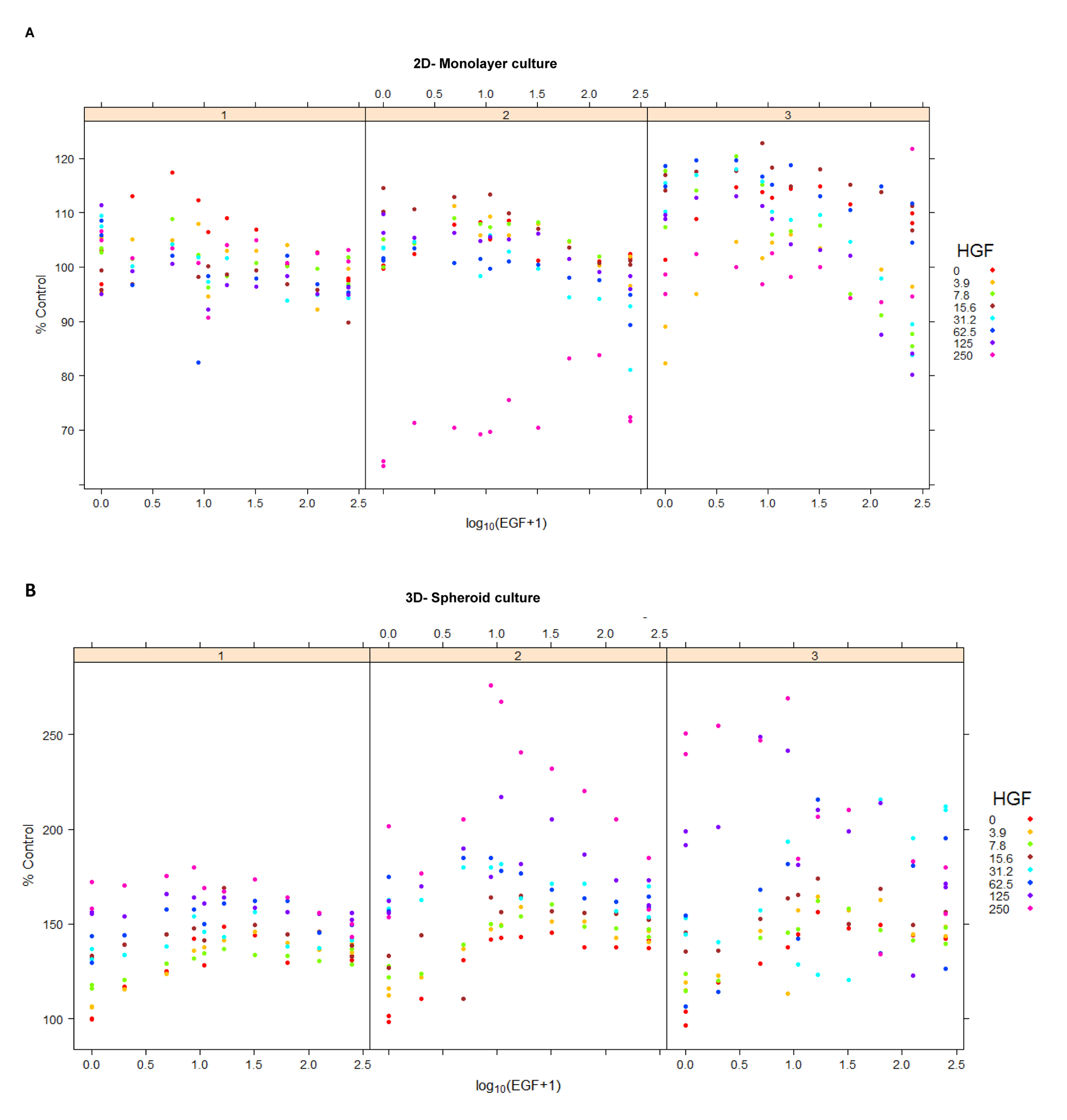

Supplement: Figure S3 — Proliferation response to EGF and HGF is altered in 3D compared to 2D. The plots for 2D (A) and 3D (B) are the growth measurements for the varying concentrations of EGF and HGF by each plate (panels). For this figure, a value of 1 is added to each original concentration value and the augmented concentration value is then transformed to the log10 scale. The y axis is growth which is a RLU value determined by CellTiter Glo after EGF and HGF for two days. (TIF) [file pone.0092248.s003.tif]

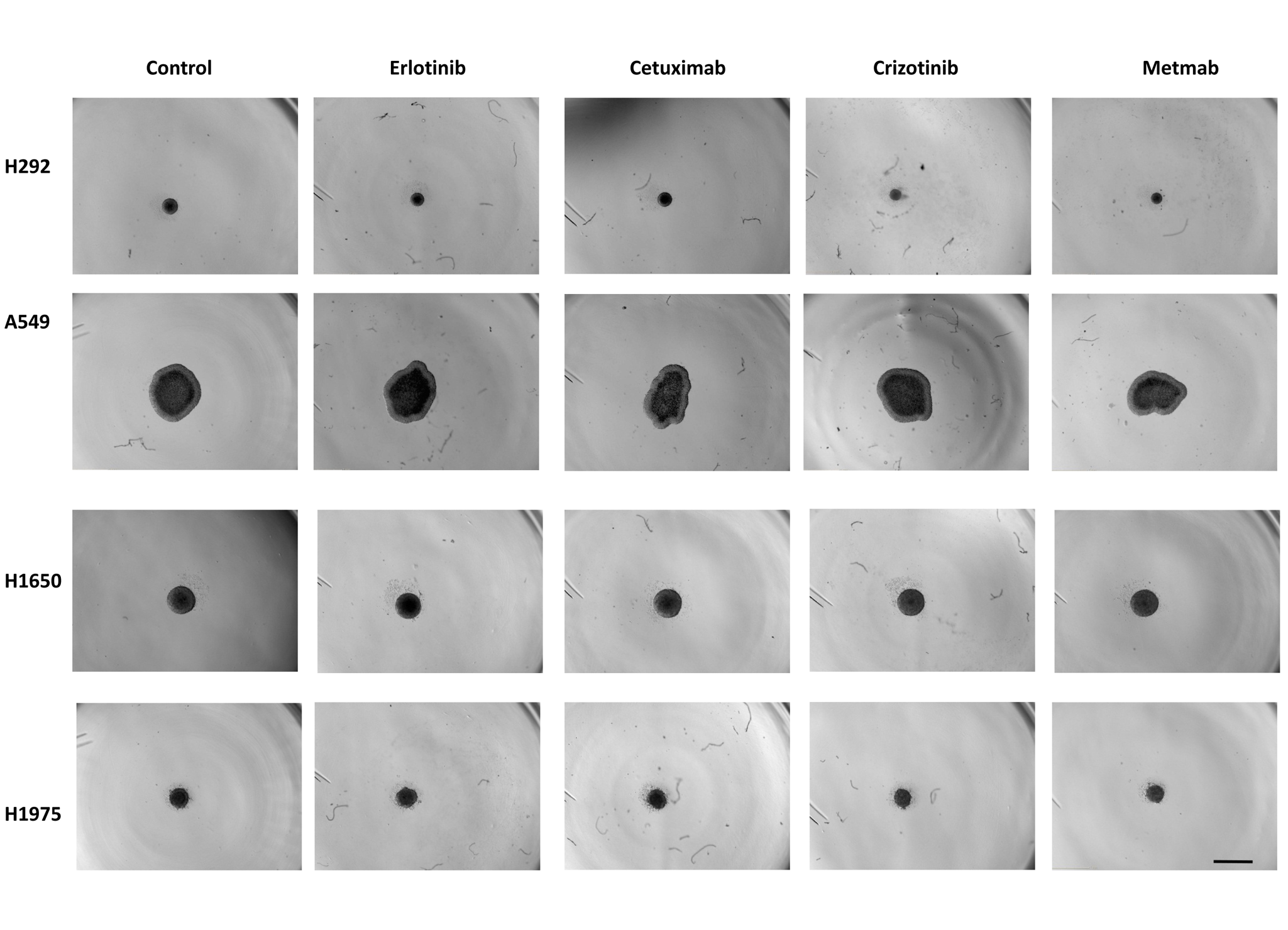

Supplement: Figure S4 — The effects of EGFR and cMET compounds in 3D spheroid proliferation. Representative bright field images showing drug response after 72 hours in 3D spheroids in cell proliferation assay. Magnification: 2x objective, scan bar 1mm. (TIF) [file pone.0092248.s004.tif]

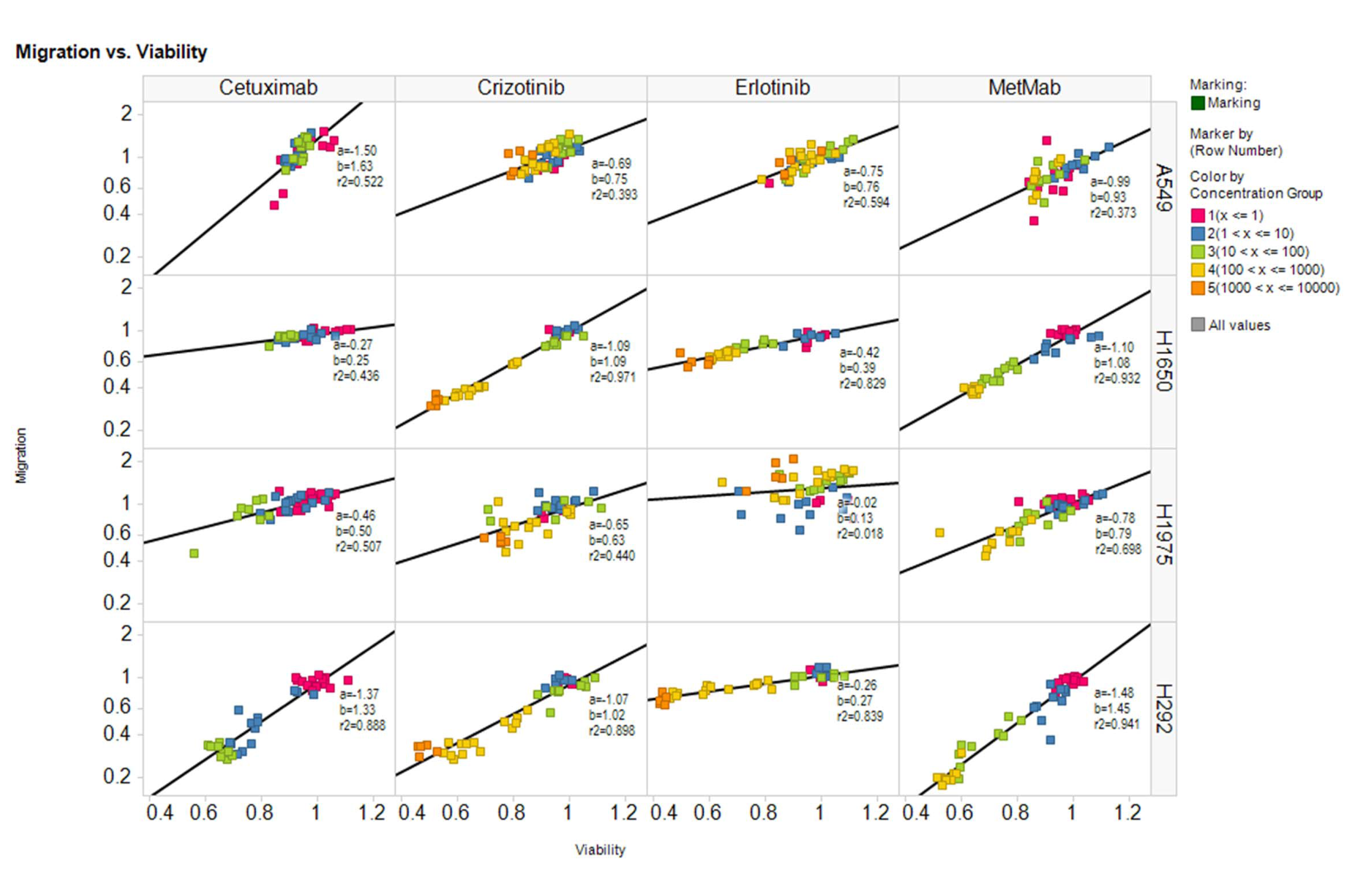

Supplement: Figure S5 — Positive correlation between cell migration and cell viability in cell migration assay. The scatterplots by cell type and compound are for log-transformed migration (total area) verses cell viability (RLU value). Total area (μm2) of migration pattern and spheroid were determined by using bright field images in a fully automated Operetta high content imaging system (Perkin Elmer). Cell viability (RLU) was determined after cell migration by CellTiter Glo. The r-squared value along with the intercept (a) and slope (b) are shown in the diagrams. (TIF) [file pone.0092248.s005.tif]
